# Artemin and an Artemin-Derived Peptide, Artefin, Induce Neuronal Survival, and Differentiation Through Ret and NCAM

**DOI:** 10.3389/fnmol.2019.00047

**Published:** 2019-02-22

**Authors:** Mirolyuba Ilieva, Janne Nielsen, Irina Korshunova, Kamil Gotfryd, Elisabeth Bock, Stanislava Pankratova, Tanja Maria Michel

**Affiliations:** ^1^Department of Psychiatry, Department of Clinical Research, University of Southern Denmark, Odense, Denmark; ^2^Psychiatry in the Region of Southern Denmark, Odense University Hospital, Odense, Denmark; ^3^Laboratory of Neural Plasticity, Department of Neuroscience, University of Copenhagen, Copenhagen, Denmark; ^4^Brain Research – Inter-Disciplinary Guided Excellence, Department of Clinical Research, University of Southern Denmark, Odense, Denmark; ^5^Research Laboratory for Stereology and Neuroscience, Bispebjerg-Frederiksberg Hospital, Copenhagen University Hospital, Copenhagen, Denmark

**Keywords:** artemin, mimetic peptides, NCAM, neuroprotection, neurite outgrowth

## Abstract

Artemin (ARTN) is a neurotrophic factor from the GDNF family ligands (GFLs) that is involved in development of the nervous system and neuronal differentiation and survival. ARTN signals through a complex receptor system consisting of the RET receptor tyrosine kinase and a glycosylphosphatidylinositol-anchored co-receptor GFL receptor α, GFRα3. We found that ARTN binds directly to neural cell adhesion molecule (NCAM) and that ARTN-induced neuritogenesis requires NCAM expression and activation of NCAM-associated signaling partners, thus corroborating that NCAM is an alternative receptor for ARTN. We designed a small peptide, artefin, that could interact with GFRα3 and demonstrated that this peptide agonist induces RET phosphorylation and mimics the biological functions of ARTN – neuroprotection and neurite outgrowth. Moreover, artefin mimicked the binding of ARTN to NCAM and required NCAM expression and activation for its neurite elongation effect, thereby suggesting that artefin represents a binding site for NCAM within ARTN. We showed that biological effects of ARTN and artefin can be inhibited by abrogation of both NCAM and RET, suggesting a more complex signaling mechanism that previously thought. As NCAM plays a significant role in neurodevelopment, regeneration, and synaptic plasticity we suggest that ARTN and its mimetics are promising candidates for treatment of neurological disorders and warrant further investigations.

## Introduction

Neurotrophic factors play an essential role in the survival, differentiation, and maintenance of neurons in the central and peripheral nervous systems (Anders et al., 2001; Enomoto et al., 2001; Honma et al., 2002). Their discovery and characterization have been instrumental for the understanding of the development, plasticity, and repair of the nervous system (Baloh et al., 2000a). The potential importance of neurotrophic factors for the development of therapeutic agents against neurodegenerative disorders and brain injury makes it vital to understand their structure, function and signaling mechanisms and may allow the design and engineering of analogs with desired pharmacological properties (Kazim and Khalid Iqbal, 2016).

Artemin (ARTN) is a member of the glial cell line-derived neurotrophic factor family ligands (GFLs) which includes three other members: glial cell line-derived neurotrophic factor (GDNF), neurturin (NRTN), and persephin (PSPN) (Baloh et al., 1998b; Airaksinen and Saarma, 2002). GFLs affect the generation, survival, and growth of neurons in different CNS neuronal populations, including midbrain dopaminergic neurons, central motor neurons, and noradrenergic neurons (Airaksinen and Saarma, 2002). Similar to GDNF, ARTN is a potential neuroprotective agent as it promotes the survival of dopaminergic neurons *in vitro* (Rosenblad et al., 2000; Sariola and Saarma, 2003). ARTN plays a role in pathogenesis and could be a target to improve the treatment of psychiatric disorders such as depression. ARTN plasma levels are reduced in patients with major depressive disorder (Pallanti et al., 2014), and intracerebroventricular administration of ARTN shows dose-dependent antidepressant effects in mice, potentially via modulation of neuronal plasticity (Mannelli et al., 2011).

Artemin also plays a role in the generation and survival of sympathetic neurons at different stages of development (Anders et al., 2001; Honma et al., 2002). Gfrα3-/- mice exhibited severe defects in the superior cervical ganglion (SCG), causing lack of sympathetic innervation in the upper eyelid and submandibular salivary gland (Nishino et al., 1999).

Systemic treatment with ARTN normalizes morphological and neurochemical properties of injured small dorsal root ganglion neurons and mitigates behavioral symptoms associated with neuropathic pain in surgically and chemically induced nerve injury models (Gardell et al., 2003; Bennett et al., 2006; Wang et al., 2008; Wong et al., 2015). Results from Phase 1 clinical trials (Rolan et al., 2015; Okkerse et al., 2016) further support the application of ARTN for treatment of peripheral nerve injury and attenuation of neuropathic pain. A recent Phase 2 trial (SPRINT) that evaluated the safety and efficacy of intravenous ARTN (neublastin, BG00010) in reducing pain in patients with lumbosacral radiculopathy showed evidence of pain relief, particularly at the lowest dose of ARTN (Backonja et al., 2017).

Artemin is a homodimer in which the two monomers are assembled in a “tail-to-head” fashion and are stabilized by an inter-chain disulfide bond (Airaksinen et al., 1999; Baloh et al., 2000a; Scott and Ibañéz, 2001).

GDNF family ligands signal through a multicomponent receptor system consisting of the RET receptor tyrosine kinase, common for all GFL members, and a ligand-specific glycosylphosphatidylinositol-anchored co-receptor GFL receptor α (GFRα_1-4_) (Airaksinen and Saarma, 2002) that determines the ligand-binding specificity of the GFRα-RET complex. ARTN specifically binds to GFRα3 (Yan et al., 2003), which is mainly expressed in the cerebellum (Masure et al., 1998). Although ARTN prefers to bind with the GFRα3-RET complex, it can also bind with the GFRα1-RET complex (Baloh et al., 1998b). Additional “cross-talk” between GFLs and GFRαs has been described (Baloh et al., 1998a; Trupp et al., 1998; Airaksinen et al., 1999). Assembling of the GFL-GFRα-RET complex triggers the dimerization of RET, leading to autophosphorylation of specific tyrosine residues in its intracellular domain and subsequent activation of different intracellular signal cascades. These include Akt, MAPK-Erk, JNK, and Src, which are involved in regulation of cell survival, differentiation, proliferation, migration, hemotaxis, morphogenesis, neurite outgrowth, and synaptic plasticity (Airaksinen and Saarma, 2002). Adding to the complexity of the system, RET is expressed in three main isoforms, of which the 3′-end alternatively spliced RET9 and RET51 are the most highly expressed and well-studied (Richardson et al., 2012). Moreover, RET9- and RET51-associated signal complexes and pathways of degradation are markedly different. The third isoform of RET, RET43, was described in humans (Carter et al., 2001). Recently, two additional functional isoforms of RET that lack either exon 3 or exons 3–5 were described in CNS (Gabreski et al., 2016).

Two alternative receptors for GDNF are described, i.e., neural adhesion molecule (NCAM) (Paratcha et al., 2003) and heparan sulfate proteoglycan syndecan-3 (Bespalov et al., 2011), which is a transmembrane proteoglycan that binds to the GFL dimer with very high affinity (Cik et al., 2000; Leppänen et al., 2004), contrary to the GFRα-RET receptor complex.

Analysis of expression profile in different brain domains, including central neocortex, cingulate cortex, basal ganglia, and hippocampus, showed that GFRαs are more widely expressed than RET (Trupp et al., 1997; Yu et al., 1998), suggesting that GFLs may signal independently of RET. This was corroborated by the finding that NCAM and GFRα1 function as an alternative signaling receptor for GDNF in hippocampal and cortical neurons (Paratcha et al., 2003). However, there have so far been no reports that NCAM and GFRα3 can act as an alternative receptor complex for ARTN.

We designed and characterized a putative ARTN mimetic peptide corresponding to the heel region, named artefin, and investigated its survival and neuritogenic potential in primary neuronal cultures, interaction with receptor complex GFRα-RET, and intracellular signaling pathways involved in the action of ARTN. We found that ARTN bound directly to NCAM, and that both NCAM expression and activation of downstream signaling partners were required for ARTN-induced neurite outgrowth, thereby indicating that NCAM is an alternative receptor for ARTN.

## Materials and Methods

### Mimetic Peptides

The sequence of ARTN-derived peptide artefin, RSPHDLSLASLLGAG (Supplementary Figure S1), corresponds to amino acids 166–180 of human ARTN (UniProt D3DPX9). The artefin peptide, its scrambled (LPLSSLRGHSGADAL) and reversed (GAGLLSALSLDHPSR) versions, and the control P2-d peptide (GRILARGEINFK) were synthesized as a tetramer composed of four monomers coupled to a lysine backbone using the solid-phase Fmoc protection chemistry (Schafer-N, Copenhagen, Denmark). The peptide purity was ≥80% as estimated by high-performance liquid chromatography. The recombinant human ARTN was purchased from R&D Systems (Abingdon, United Kingdom).

### Circular Dichroism Spectroscopy

Circular dichroism (CD) spectra were measured over the range 190–250 nm using a Jasco J-810 spectropolarimeter in cells with path length 1 mm at room temperature. The peptide was dissolved in 10 mM NaH_2_PO_4_ buffer, pH 7.0, to the final concentration 10 μM. Data were recorded at a scan speed of 20 nm/min with 10 repeat scans accumulated to obtain the final average spectra. Following the buffer subtraction, the observed ellipticity θ (mdeg) was converted to mean residue ellipticity [θ] (deg cm^2^/dmol) using the following relationship [θ] = 100θ /(*lcn*) where ‘*l*’ is path length in centimeters, ‘*c*’ is the millimolar concentration, and ‘*n*’ is the number of residues in the peptide.

### Cell Cultures

#### Primary Cultures of Cerebellar Granule Neurons

Cerebellar granule neurons (CGNs) were prepared from 7-day-old Wistar rat pups (Charles River Laboratories, Sulzfeld, Germany) as previously described (Schousboe et al., 1989). Briefly, pups were decapitated, and the cerebellum was removed and cleared from blood vessels and meninges in ice-cold modified Krebs-Ringer buffer. The cerebellum was dissociated by chopping and trypsinization. Cells were washed in Krebs-Ringer buffer containing DNAse I (Sigma, St. Louis, MO, United States) and soybean trypsin inhibitor (Sigma) to stop trypsinization, and the remaining tissue pieces were pelleted by centrifugation. Cells were then washed in Krebs-Ringer buffer containing Ca^2+^ and Mg^2+^ and re-suspended in appropriate Neurobasal^TM^ medium (Invitrogen, Taastrup, Denmark) with supplements depending on the experiment.

#### Cell Lines

The pheochromocytoma cell line PC12-E2 (gift from Dr. Klaus Seedorf, Hagedorn Research Institute, Gentofte Municipality, Denmark) was propagated in Dulbecco’s modified Eagle’s medium (DMEM) supplemented with 5% (v/v) fetal calf serum (FCS), 10% (v/v) horse serum (HS), 1% (v/v) glutamax, 100 U/ml penicillin, and 100 μg/ml streptomycin. Cells were grown at 37°C in a humidified atmosphere containing 5% CO_2_.

### Survival Assay

Cerebellar granule neurons were re-suspended in Neurobasal^TM^-A medium supplemented with 2% (v/v) B27 (Invitrogen, Taastrup, Denmark), 0.5% (v/v) glutamax, 100 U/ml penicillin, 100 μg/ml streptomycin, and 40 mM KCl, and seeded at a density of 1 × 10^5^ cells/well in 8-well LabTek Permanox chamber slides (Nunc, Roskilde, Denmark) coated with poly-L-lysine (10 μg/ml). To avoid proliferation of non-neuronal cells, cytosine-β-D-arabinofuranoside (Sigma) was added to cells 24 h after plating. CGNs were allowed to differentiate for 7 days *in vitro* (DIV) in the presence of 40 mM KCl before being induced to undergo apoptosis by changing the medium to one containing only 5 mM KCl (apoptotic medium) (D’Mello et al., 1993). At 7 DIV, cells were washed and stimulated with serially diluted ARTN or artefin diluted in the apoptotic medium. Three different controls were included; (1) cells grown in medium containing 40 mM KCl served as a positive control; (2) cells grown in medium containing 5 mM KCl served as a negative control; (3) cells grown in medium containing 5 mM KCl supplemented with 50 ng/ml insulin growth factor-1 (IGF-1) (Life Technology), a known anti-apoptotic factor for CGNs in this setup (Galli et al., 1995). After 48 h following the induction of apoptosis, cells were fixated in 4% formaldehyde for 30 min, and nuclear morphology was visualized with Hoechst 33258 staining (Invitrogen, Taastrup, Denmark) (Figure 2A). Images of cells were obtained using a Nikon Diaphot 200 fluorescent microscope equipped with a Nikon Plan 40× objective (Nikon, Tokyo, Japan) and coupled to a black-white video camera. Images of at least 1000 cells/well in different fields of view were analyzed, and the percentage of viable neurons was estimated as the ratio of live cells (non-pyknotic cells with dispersed chromatin) to the total number of neurons.

### Neurite Outgrowth Assay

Cerebellar granule neurons CGNs were re-suspended in Neurobasal^TM^ medium supplemented with 2% (v/v) B27, 0.5% (v/v) glutamax, 100 U/ml penicillin, 100 μg/ml streptomycin, 0.4% (w/v) BSA, and 20 mM HEPES and were seeded in 8-well LabTek Permanox chamber slides (Nunc) at a density of 1 × 10^4^ cells/cm^2^. Various concentrations of ARTN, artefin, scrambled artefin, or reversed artefin were added to neurons immediately after seeding. For the RET inhibition assay, the inhibitory goat anti-mouse RET antibody (4.1 μg/ml; R&D systems, Abingdon, United Kingdom) or control goat IgG (4.1 μg/ml; Santa Cruz Biotechnology, Dallas, TX, United States) were added to neurons 1 h before stimulation with ARTN (0.042 nM) or artefin (4.2 μM). For the competition assay between growth factor and mimetic peptide, a combination of ARTN (2.1 nM) and artefin (4.2 μM) was added to the cells immediately after seeding. To inhibit Fibroblast growth factor receptor (FGFR) pharmacologically, inhibitor of FGFR, SU5402 (Sigma), was added to the growth medium in serial dilutions of 20, 40, and 80 μM immediately after plating of CGNs, followed by addition of either P2-d (8 μg/ml), ARTN (0.21 nM), or artefin (1.4 μM) 1 h after the plating.

To evaluate the involvement of NCAM and RET in neurite outgrowth induced by either ARTN or artefin, CGNs (3 × 10^6^ per transfection) were transfected with 3 μg DNA using a Nucleofector^TM^ 2b device and a Rat Neuron Nucleofector Kit (Amaxa, Inc., Gaithersburg, MD, United States). A kinase-deleted dominant negative RET insert was obtained by direct PCR using a commercially available clone (IRAKp961P02132Q; RZPD, Berlin, Germany) encoding full-length mouse RET coding sequence 586–3807 nt. as template. The upper primer ATATATGCTAGCTATGGCGAAAGCGACGTCCGG contained a NheI restriction site and Kozak sequence, and the lower primer ATATATGCGGCCGCTTATTTTTCGAACTGCGGGTGGCTCCAAGCGCTAACCTGGTTCTCCGTGGAATCCAG encoded Streptag II and NotI restriction sites. The dominant negative RET insert and the pcDNA5/FRT expression vector containing the Flp-In system were cut with NheI and NotI restriction enzymes, purified, ligated using the Rapid DNA Ligation Kit (Roche, Basel, Switzerland), and transfected into One Shot TOP10 *E. coli* (Invitrogen, Taastrup, Denmark). *E. coli* colonies containing the dominant negative RET insert were identified by PCR, and one insert-positive colony was used for plasmid purification. The identity of the purified plasmid was verified by restriction cutting and sequencing of the insert. For NCAM knock down, we used a pENTR vector containing the shRNA expression cassette for NCAM knock down (Hansen et al., 2007). An empty vector was used as a control. To identify transfected neurons, all transfections were performed as co-transfections with 0.5 μg pEGFP-N1, an expression vector encoding the enhanced variant of green fluorescent protein (GFP; Clontech, Palo Alto, CA, United States). Transfected CGNs were seeded in Neurobasal^TM^-A medium supplemented with 2% (v/v) B27, 5% (v/v) FCS, and 2 mM Glutamax at a density of 1 × 10^5^ cells/cm^2^.

To inhibit FGFR function in neurons, CGNs (3 × 10^6^) were transfected with either a vector encoding a dominant negative version of FGFR1 (dnFGFR), lacking the kinase domain (Saffell et al., 1997; Enevoldsen et al., 2012) or the corresponding empty control vector using Amaxa Nucleofection (Lonza, Cologne, Germany). The pEGFP-N1 was added as a transfection control as previously described (Enevoldsen et al., 2012). The transfected cells were plated at a density of 5 × 10^5^ cells/well in transfection medium [Neurobasal^TM^-A medium supplemented with 2% (v/v) B27, 2% (v/v) horse serum, and 2 mM glutamax].

To estimate neurite outgrowth, neurons were fixated 24 h after stimulation and stained with rabbit anti-growth-associated-protein-43 (GAP-43) antibody (Chemicon International, Inc., Temecula, CA, United States) or anti-GFP antibody (for transfection experiment) overnight at 4°C followed by incubation with secondary Alexa-conjugated goat anti-rabbit antibodies (Invitrogen, Taastrup, Denmark). In case of experiments with plasmid transfection, the transfection efficiency was ca. 20% of total cells and the neurite outgrowth was evaluated only in GFP-positive, i.e., transfected, cells. For representative pictures, cells were additionally co-stained with Hoechst 33258.

Computer-assisted fluorescent microscopy was performed using a Nikon Diaphot 300 inverted microscope (Nikon) equipped with a Nikon Plan 20× objective and coupled to a video camera (Grundig Electronics, Germany). For each condition in each experiment, including the experiment with transfections, images of approximately 200 cells were recorded in systematic series of fields of view, and neurite outgrowth was quantified using a stereological approach as previously described (Rønn et al., 2000).

### Surface Plasmon Resonance (SPR) Analysis

The binding analysis was performed on a Biacore^TM^ 2000 instrument (GE Healthcare, Hillerød, Denmark) at 25°C using HBS-EP (10 mM HEPES pH 7.4, 150 mM NaCl, 3 mM EDTA, and 0.005% v/v Surfactant P20) as running buffer. ARTN and artefin were immobilized on a CM 4 sensor chip at a flow rate of 5 μl/min using amine coupling kit (GE Healthcare). The chip was activated with 35 μl activation solution containing *N*-hydroxysuccinimide (NHS) and *N*-ethyl-*N*′-(dimethylaminopropyl)-carbodiimide (EDC). Approximately 35 μl 2.1 nM ARTN in 10 mM sodium acetate (pH 5.0) or 35 μl 78 μM artefin in 10 mM sodium acetate (pH 5.0) were injected over the chip yielding immobilization levels of approximately 2300 and 1600 RU, respectively. The chip was then deactivated with 35 μl 1.0 M ethanolamine. For binding analysis, serially diluted recombinant human GFRα3/Fc (R&D Systems, Abingdon, United Kingdom) and recombinant rat NCAM Ig1–3, produced as described previously (Soroka et al., 2003), were diluted in HBS-EP buffer and injected over the chip at a flow rate of 20 μl/min. Regeneration was performed with 150 mM NaCl containing 12.5 mM NaOH (for GFRα3) or 2 M NaCl (for Ig1–3). In reverse binding experiments, GFRa1/Fc and GFRa2/Fc (0.113 μM; R&D systems) were immobilized on CM 4 chip and first the corresponding positive control proteins, GDNF (5.07 nM) and NRTN (6.2 nM), were injected. The binding of ARTN (6.2 nM) and artefin (15.6 μM) were performed simultaneously. The curves corresponding to the differences between the binding to the immobilized protein and the binding to a blank well were used for analysis. Curves were further referenced by subtracting appropriate control curves obtained by injecting HBS-EP buffer alone. Curves were analyzed by non-linear curve fitting using a 1:1 interaction model or steady-state affinity analysis applying the software package BIAevaluation v. 4.1 (GE Healthcare).

### RET Phosphorylation Assay

Phosphorylation of RET was assayed in PC12-E2 cells. Cells were seeded at a density of 7 × 10^4^ cells/cm^2^ in 6 cm cell culture dishes (Nunc) and grown for 24 h. Cells were then grown in starvation medium (growth media with FCS switched to 1%) for 4 h before being stimulated with recombinant human ARTN (2.1 nM) or artefin (12.64 μM) for 10 min. Non-stimulated cells served as a control. Cell lysates were separated by SDS-polyacrylamide gel electrophoresis (SDS-PAGE) and transferred to an Immobilon-P membrane (Millipore, Billerica, MA, United States). Membranes were stained with mouse anti-phosphotyrosine antibody (BD Transduction Lab, New York City, NY, United States; 1:500) or RET (goat anti-mouse antibody, R&D systems, 1:500), followed by incubation with HRP-conjugated anti-mouse IgG. Immune complexes were visualized by chemiluminescence (SuperSignal West Dura Extended Duration Substrate, Pierce Biotechnology, Rockford, IL, United States) using a GeneGnome (Syngene, Cambridge, United Kingdom). Membranes were stripped for immune complexes and re-probed using goat anti-RET antibody (R&D systems; 1:500) followed by incubation with HRP-conjugated rabbit anti-goat IgG. RET phosphorylation was determined as the ratio between phosphorylated and total RET.

### Statistical Analysis

Results are expressed as mean values ± standard error of the mean (SEM). The data are given as percentage of control, the control being set to 100%. Statistical evaluation of data was performed using either paired *t*-test or one-way analysis of variance (ANOVA) for repeated measurements followed by Dunnett’s or Tukey’s post-tests. Evaluation was done using the software package GraphPad Prism v.4.02 (GraphPad Software, Inc., San Diego, CA, United States) or FIG-P version 2.98 (Biosoft, Cambridge, United Kingdom). Asterisks indicate the significance levels as follows: ^∗^*p* < 0.05; ^∗∗^*p* < 0.01; ^∗∗∗^*p* < 0.001; ^∗∗∗∗^*p* < 0.0001.

## Results

### ARTN and an ARTN-Derived Peptide, Artefin, Promote the Survival of CGNs

**FIGURE 1:**
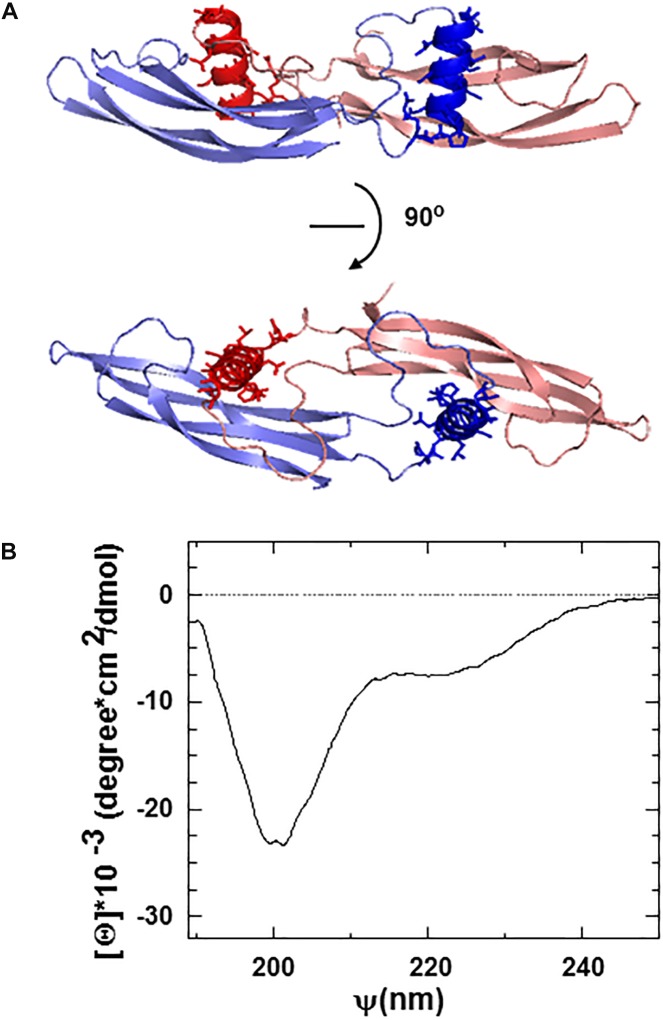
**(A)** Ribbon structure of the ARTN dimer indicating the position of artefin sequence (shown in dark red and dark blue) corresponding to the heel region of the molecules. **(B)** Circular dichroism (CD) spectra of the artefin tetrameric peptide. The minimum at 208 nm, a shoulder at 222 nm, and a pick at 193 nm indicate that artefin keeps the a-helical conformation in solution.

The ARTN monomer is composed of two β-sheet fingers, a cystine-knot core motif, and an α-helical heel region (Figure 1A). Finger 1 comprises two long continuous antiparallel β strands, whereas finger 2 has interruptions in the middle, resulting in five relatively short β-strands in the β-sheet. Within the dimer, the helix in the heel region of one ARTN monomer contacts the finger region of another monomer with its helical axis nearly perpendicular to the β-strands [43, 44, and Figure 1A].

We focused on the α-helical heel region of ARTN, where a number of side chains are exposed outside of the dimer and thus available for potential interactions with other molecules. We designed a peptide covering this part of the molecule and named it artefin (Figure 1A). Far-ultraviolet CD spectroscopy of artefin peptide showed a negative band at 208 nm, a shoulder at 222 nm, and a positive band at 193 nm (Figure 1B), indicating that a-helix confirmation is dominant for the artefin peptide in solution.

**FIGURE 2:**
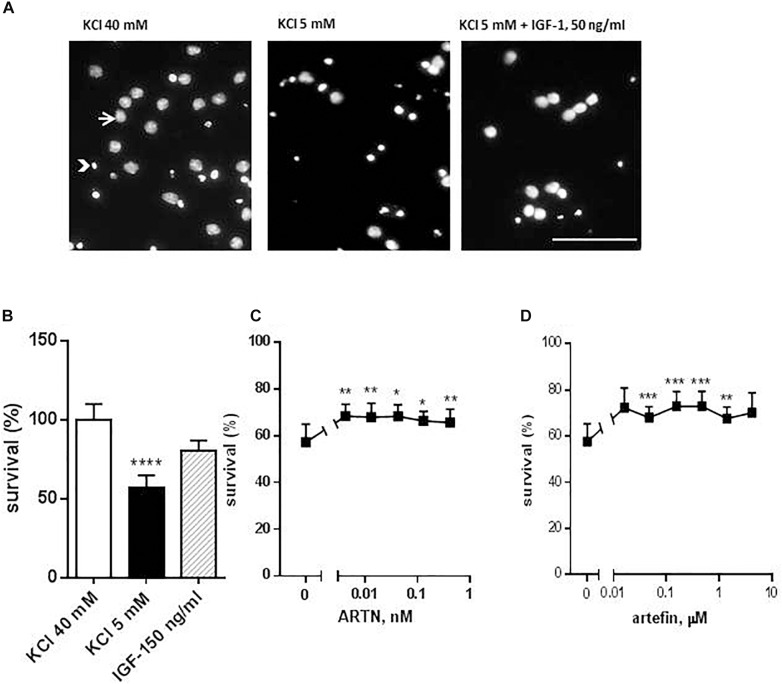
Effect of ARTN and artefin on survival of cerebellar granular neurons (CGNs) induced to undergo apoptosis. CGNs were differentiated for 7 days in the presence of 40 mM KCl before apoptosis was induced by potassium withdrawal. **(A)** Nuclear morphology of CGNs after Hoechst 33258 staining. The arrow shows the intact nuclei with dispersed chromatin and arrowhead – picnotic nuclei with condense chromatin. Scale bar: 100 μm. Cells were grown for 48 h in media, containing 40 mM KCl, apoptotic medium (5 mM KCl) or apoptotic medium supplemented with 50 ng/ml IGF-1 (positive control) **(B)**, serially diluted ARTN **(C)**, or artefin **(D)**. Each experiment was performed in the presence of the three controls (40 mM KCl, 5 mM KCl, and 5 mM KCl plus IGF-1), and the survival effect of the growth factor and the peptide was compared to its own apoptotic control (5 mM KCl). Results from the five experiments are expressed as percentage of 40 mM KCl control set at 100% and presented as mean ± SEM. The anti-apoptotic effect of ARTN and artefin was tested using Student’s paired *t*-test, where ^∗^*p* < 0.05; ^∗∗^*p* < 0.01; ^∗∗∗^*p* < 0.001; ^∗∗∗∗^*p* < 0.0001.

The neuroprotective function of ARTN is well-known, and ARTN promotes the survival of various central and peripheral neuronal populations (Anders et al., 2001; Enomoto et al., 2001; Honma et al., 2002). Therefore, to investigate the putative mimetic abilities of artefin, we tested whether artefin promotes neuronal survival *in vitro* using CGNs, which are known to express GFRα1, GFRα3, and RET (Nosrat et al., 1997; Widenfalk et al., 1997; Masure et al., 1998). As CGNs require depolarizing concentrations of KCl for survival and differentiating *in vitro* (D’Mello et al., 1993; Widenfalk et al., 1997), the change to the apoptotic medium with low potassium led to a significant decrease in the proportion of live cells (Figure 2A,B, black column vs. white column). Treatment with 50 ng/ml IGF-1, which is a known pro-survival factor for CGNs both *in vitro* (Galli et al., 1995) and *in vivo* (Chrysis et al., 2001), significantly increased the number of CGNs rescued from the apoptosis induced by the potassium withdrawal (*p* < 0.0001; Figure 2A,B).

Addition of serially diluted ARTN to apoptotic medium promoted the survival of CGNs in culture in a dose-dependent manner (Figure 2C). The maximal neuroprotective effect (68.3% of control) was obtained with 0.004 nM ARTN. We subsequently investigated the effect of artefin and found that serially diluted peptide significantly promoted neuronal survival (Figure 2D).

The maximum level of neuronal survival was 72.95% of the controls and was obtained with 0.156 μM artefin. These data show that artefin can mimic the neuroprotective function of ARTN, indicating that artefin may be a functional ARTN mimetic. Of note, ARTN and artefin have similar efficacy (neuroprotective effect), but the peptide has a lower potency as its effect is in the range of μM concentrations while ARTN works in the nM range.

### ARTN and Artefin Induce Neurite Outgrowth in CGNs

**FIGURE 3:**
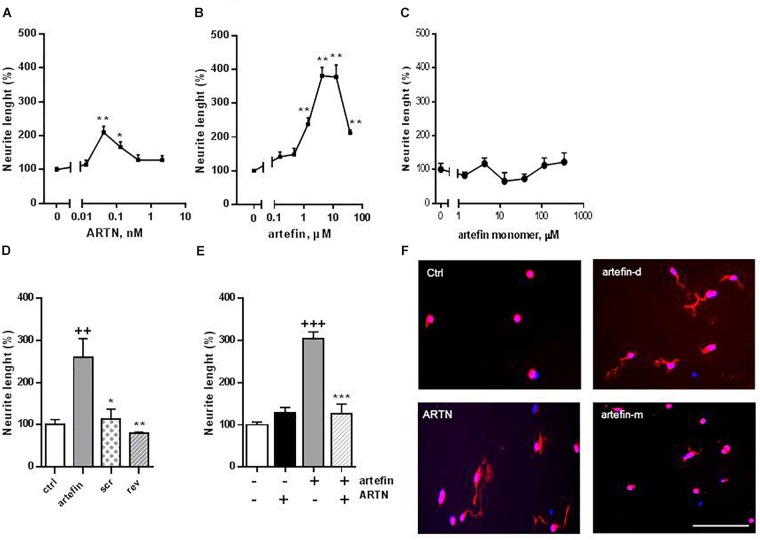
Neurite outgrowth induced by ARTN **(A)**, artefin-dendrimer **(B)**, and artefin-monomer **(C)** in CGNs. Cells were stimulated with ARTN (0.013, 0.042, 0.13, 0.42, and 2.1 nM) or artefin (0.16, 0.47, 1.4, 4.2, 12.6, and 37.9 μM) for 24 h. For the unstimulated control, the absolute length of neurites was 11.47 ± 1.5 μm. Results from four independent experiments are expressed as percentage of untreated control set to 100% and presented as mean ± SEM. The mean neurite outgrowth lengths were compared using one-way ANOVA for repeated measurements. The effect of individual concentrations of ARTN and artefin was compared to control using one-way ANOVA with Dunnett’s post-hoc test, ^∗^*p* < 0.05; ^∗∗^*p* < 0.01. **(D)** Neuritogenic effect of artefin, scrambled and reverse peptides (tetramers). CGN neurons were treated with peptides in concentration of 4.2 μM for 24 h. The results are expressed as percentage of untreated control set to 100% and presented as mean ± SEM. The neuritogenic effect was compared using Student’s paired *t*-test where + indicates *p*-values for comparison to the negative control of untreated neurons, and ^∗^ indicates *p*-values for comparison to the positive control of neurons treated with artefin. ^++^*p* < 0.01; ^∗^*p* < 0.05, ^∗∗^*p* < 0.01. **(E)** Inhibition effect of ARTN on neurite outgrowth, by competing with artefin. CGNs were grown for 24 h in the presence of none-neuritogenic concentration of ARTN (2.1 nM), artefin (4.2 μM), or co-incubated with ARTN together with artefin in the same concentrations. Results are expressed as percentage of untreated control set at 100% and presented as mean ± SEM. The neuritogenic effect was compared using Student’s paired *t*-test, where + indicates *p*-values for comparison to the negative control and ^∗^ indicates *p*-values for comparison to neurons stimulated with artefin alone. ^+++^*p* < 0.001; ^∗∗∗^*p* < 0.001. **(F)** Representative images of CGNs stimulated with ARTN (0.042 nM), artefin-dendrimeric (atrefin-d; 4.2 μM) and artefin-monomer (artefin-m; 4.2 μM) double-stained for GAP-43 (red) and Hoechst (blue). Scale bar: 100 μm.

Neuritogenesis is a key process for proper development of the nervous system, and neurotrophic factors are known to modulate neurite outgrowth (Yan et al., 2003). ARTN has been shown to induce neurite outgrowth from a number of neuronal populations, e.g., dorsal root and superior cervical and lumbar sympathetic ganglia (Anders et al., 2001; Enomoto et al., 2001; Honma et al., 2002; Yan et al., 2003). Therefore, we aimed to investigate whether artefin could also mimic neurite outgrowth induced by ARTN. ARTN induced neurite outgrowth from primary CGNs in a bell-shaped dose-response manner (Figure 3A), and the overall neuritogenic effect of ARTN was statistically significant (*F* = 5.069, *p* < 0.01). The maximum level of neurite outgrowth was 210% of the controls and was obtained with 0.042 nM ARTN (Figure 3F). Subsequent treatment with artefin, applied in a form of dendrimer, also induced neurite outgrowth in CGNs (Figure 3B) and was actually more effective (higher length of neurites) than ARTN. The overall neurite elongation effect of artefin was statistically significant (*F* = 9.723, *p* < 0.0001), and the maximum effect (404% of control) was obtained with 4.2 μM of artefin (Figure 3F). In contrast, the artefin in monomer form did not demonstrate any significant neurite outgrowth in CGNs (Figure 3C,F).

To ensure the specificity of the mimetic effect of artefin, we tested the neurite outgrowth potential of a scrambled and a reversed versions of artefin (designed as tetramers) (Figure 3D). Artefin-treated neurons exhibited a statistically significant increase in neurite length when compared to the control condition (*p* < 0.01). In contrast, the level of neurite outgrowth was similar to control for neurons treated with either the scrambled or reversed version of the artefin peptides, thereby confirming the sequence-specific neurite outgrowth effect of artefin.

To further substantiate our finding, we tested whether artefin uses the same receptor complex as ARTN, i.e., whether a high, non-neuritogenic concentration of ARTN could impede the neurite outgrowth effect of artefin (Figure 3E). Neurite outgrowth in neurons treated with high concentration of ARTN alone was equivalent to that of the control condition, whereas neurons treated with artefin alone showed a significant increase in neurite outgrowth (*p* < 0.001). However, when neurons were co-incubated with artefin in the presence of ARTN, the neurite outgrowth effect of artefin was eliminated (*p* < 0.001; Figure 3E), indicating that artefin and ARTN compete for the same receptor complex.

### ARTN and Artefin Bind to GFRα3

**FIGURE 4:**
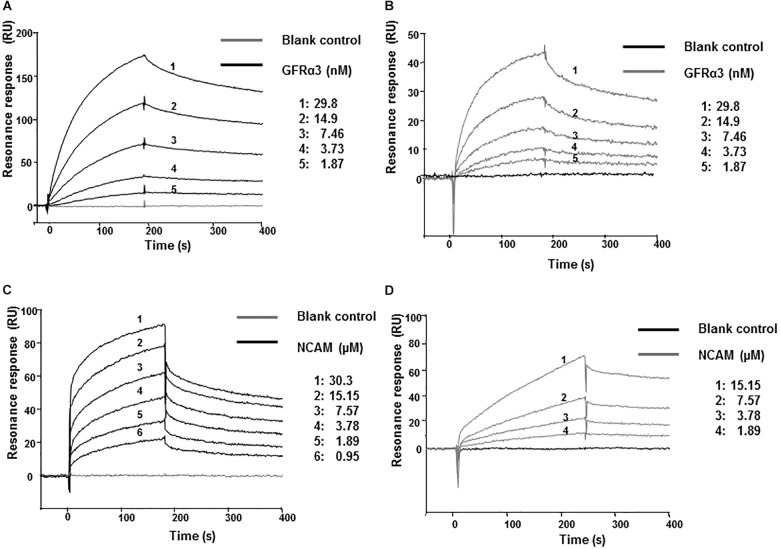
Surface plasmon resonance (SPR) analysis of binding of immobilized ARTN or artefin to serially diluted recombinant human GFRα3/Fc **(A,B)** and ARTN or artefin to serially diluted recombinant rat NCAM Ig1–3 domain **(C,D)**. Immobilization levels of approximately 2300 RU for ARTN and 1600 RU for artefin were reached. Binding curves were analyzed by non-linear curve fitting using a 1:1 interaction model or steady-state affinity analysis applying the software package BIA evaluation v. 4.1 (GE Healthcare).

We next investigated whether artefin directly binds to GFRα3 by surface plasmon resonance (SPR). To confirm the proper functioning of GFRα3, its known ligand, ARTN, was first used in binding experiments as a positive control. GFRα3 showed a clear concentration-dependent binding to ARTN (Figure 4A), and the affinity constant *K_D_* for the ligand–receptor interaction was in the low nanomolar range (Table 1).

**Table 1:** Affinities for ligand-receptor interactions.

Injected protein	*ARTN, K_D_ (M)*	*Artefin, K_D_ (M)*
GFRα3	2.7 × 10^-9^ ± 2.8 × 10^-10^	2.9 × 10^-9^ ± 6.5 × 10^-10^
NCAM	1.3 × 10^-5^ ± 3.4 × 10^-6^	2.8 × 10^-6^ ± 7.9 × 10^-7^


*Curves were analyzed by non-linear curve fitting using a Langmuir equation for 1:1 binding. The binding affinities were calculated from three independent experiments and are given as the mean ± SEM.*

GFRα3 also showed a clear, concentration-dependent binding to artefin (Figure 4B), and the affinity of the interaction was similar to that of the ARTN-GFRα3 interaction (Table 1). Moreover, similar to ARTN, which has previously been shown to interact with GFRα1and GFRα2 (Scott and Ibañéz, 2001), artefin binds to GFRα1 and GFRα2, suggesting that artefin is a promiscuous binding epitope within ARTN sequence (Supplementary Figure S2).

### ARTN and Artefin Bind Directly to NCAM

The involvement of NCAM in ARTN-induced effects has not been reported previously, although studies indicate that ARTN-related GDNF can bind and signal through NCAM (Poteryaev et al., 1999; Trupp et al., 1999; Paratcha et al., 2003). We investigated whether NCAM can also be a functional receptor for ARTN. The recombinant NCAM protein comprising Ig1–3 domains, showed a clear, concentration-dependent binding to ARTN in SPR analysis (Figure 4C), indicating that there is a direct interaction between ARTN and NCAM. The *K_D_* for the interaction was in the low micromolar range (Table 1). These results indicate that ARTN can utilize NCAM as a receptor.

We have previously shown that the GDNF-binding site for NCAM is localized to the heel region and that gliafin, a GDNF-derived peptide covering this region, binds to NCAM (Nielsen et al., 2009). As the tertiary structure of ARTN is similar to that of GDNF, we speculated that the artefin motif also contains a potential binding site for NCAM. As shown in Figure 4D, NCAM binds to artefin with the *K_D_* in the low micromolar range (Table 1), supporting the suggestion that artefin represents a binding site for NCAM within the heel region of ARTN.

### ARTN and Artefin Induce Phosphorylation of RET

**FIGURE 5:**
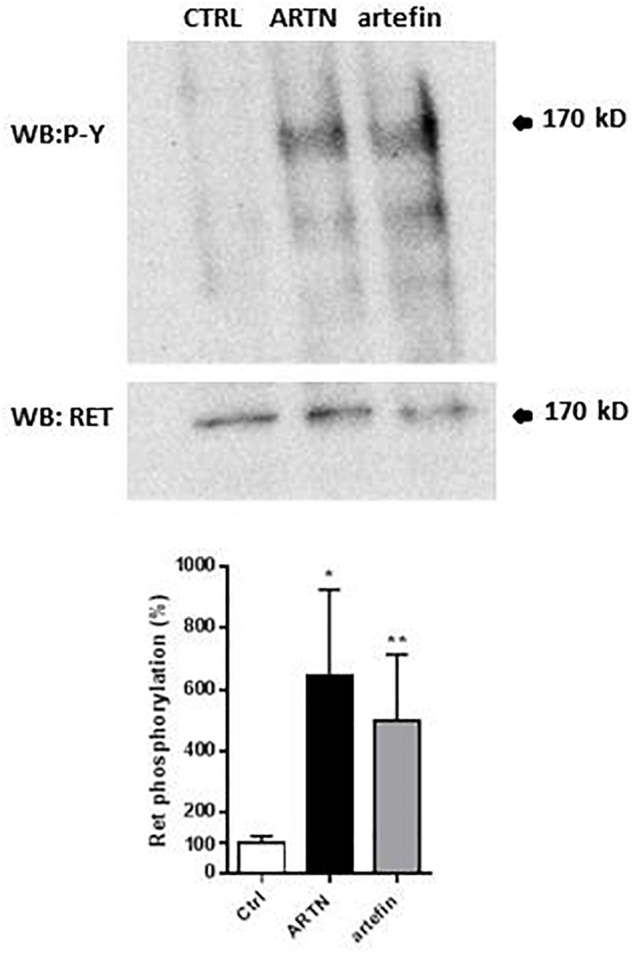
Artemin- and artefin-induced phosphorylation of RET receptor. Serum-starved PC12-E2 cells were stimulated with 2.1 nM ARTN or 12.64 μM artefin for 10 min. Non-treated cells served as control. Lysates were prepared and examined by immunoblot for phosphotyrosine and total RET after stripping of the membrane. The bar diagram shows the phosphorylation expressed as a ratio between phosphorylated tyrosine residues and amount of total RET. The results are shown as percentage of control set at 100% and presented as mean ± SEM. The asterisks indicate the significance levels: ^∗^*p* < 0.05; ^∗∗^*p* < 0.01.

The binding of artefin to GFRα3 suggests that artefin may utilize a GFRα3-RET receptor complex, and we therefore tested if artefin could induce RET activation. Stimulation of PC12 cells with ARTN, serving as a positive control, led to a clear, statistically significant increase in RET phosphorylation [∼600% compared to unstimulated control cultures (Figure 5)]. Artefin also significantly stimulated RET phosphorylation and had a similar efficiency to ARTN (∼500%).

### ARTN- and Artefin-Induced Neurite Outgrowth Involves RET

**FIGURE 6:**
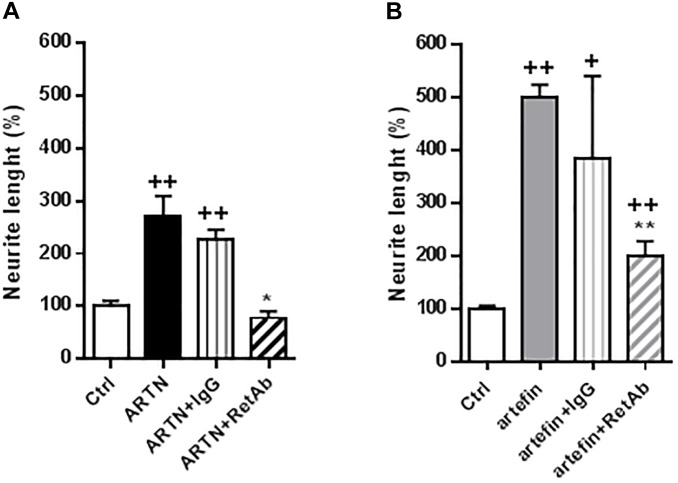
Inhibition of ARTN-induced **(A)** and artefin-induced **(B)** neurite outgrowth in CGNs by anti-RET antibody. CGNs were pre-treated directly after plating for 1 h with anti-RET antibody or control IgG in concentrations 4.1 μg/ml before stimulating with ARTN (0.042 nM) or artefin (0.47 μM). Student’s paired *t*-test was used for statistical evaluation of the results. + Indicates *p*-values for comparison of the neuritogenic effect of ARTN or artefin to the untreated control set at 100%, while ^∗^ shows *p*-values for the comparison of the inhibition effect of RET antibody to the control IgG. ^+^*p* < 0.05; ^++^*p* < 0.01; ^∗^*p* < 0.05; ^∗∗^*p* < 0.01.

To investigate if the functional effects of artefin could be mediated via RET, we tested whether anti-RET antibodies would reduce artefin-induced neurite outgrowth. Neurons stimulated with ARTN (Figure 6A) had a significantly higher level of neurite outgrowth than control cultures. Incubation with control IgG did not affect the ARTN-induced neurite outgrowth, but anti-RET antibodies completely abolished the neurite elongation effect of ARTN. When neurons were stimulated with artefin (Figure 6B), neurite outgrowth was, as expected, significantly increased when compared to the control (*p* < 0.01). Co-incubation with control IgG did not significantly alter the neuritogenic effect of artefin, whereas co-incubation with anti-RET antibodies led to a statistically significant reduction of artefin-induced neurite outgrowth (*p* < 0.01 versus artefin alone). Of note, the artefin-induced neurite outgrowth was not completely abrogated by anti-RET antibody, and was still significantly different from unstimulated control (*p* < 0.01, Figure 6B). This shows that artefin-induced neurite outgrowth involves RET, similar to ARTN and suggesting that the neurite outgrowth effect of artefin may also be mediated via other receptors.

**FIGURE 7:**
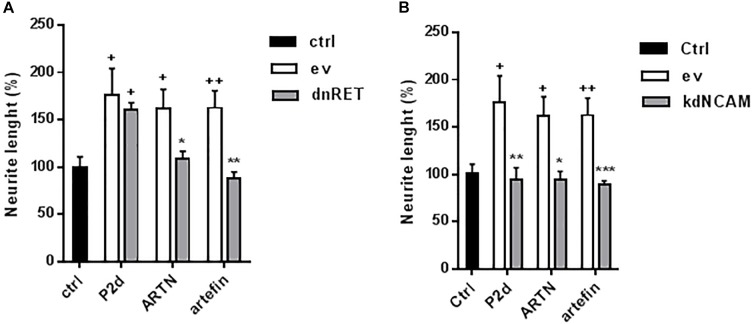
Neuritogenic effect of ARTN and artefin in **(A)** dominant negative RET or **(B)** knock down NCAM-expressing CGNs. CGNs were transfected with empty vector (ev), dominant negative version of RET (dnRet), or shNCAM-encoding plasmid (kdNCAM) and stimulated with P2-d (8 μg/ml), ARTN (0.21 nM), and artefin (1.4 μM) for 24 h. The results are presented as percentage of control set at 100%, and error bars indicate SEM. Student’s paired *t*-test was used for evaluation. + Presents *p*-values for comparison of the neuritogenic effect of compounds to the untreated neurons, while ^∗^ shows *p*-values for comparison to the positive control of CGNs transfected with empty vector. ^+^*p* < 0.05; ^++^*p* < 0.01; ^∗^*p* < 0.05; ^∗∗^*p* < 0.01; ^∗∗∗^*p* < 0.001.

To further test the involvement of RET in artefin-induced neurite outgrowth, we transfected CGNs with a kinase-deleted dominant negative version of RET (dnRET) before stimulating neurons with ARTN or artefin. As a control, we stimulated transfected cells with the NCAM-derived P2-d-peptide; its sequence was derived from the NCAM homophilic binding site, and it is known to stimulate neurite outgrowth through NCAM (Jensen et al., 1999; Kasper et al., 2000; Soroka et al., 2003). Thus, P2-d-induced neurite outgrowth should not be affected by expression of dnRET. In line with this, we found that P2-d significantly increased the level of neurite outgrowth in mock-transfected neurons as well as in neurons transfected with the vector encoding dnRET (*p* < 0.05; Figure 7A). Neurite outgrowth was significantly increased by ARTN in mock-transfected neurons (*p* < 0.05 versus control), whereas ARTN-induced neurite outgrowth was abolished in neurons expressing dnRET (*p* < 0.05 versus ARTN alone; Figure 7A). Similarly to its parent protein, artefin significantly increased neurite outgrowth in mock-transfected neurons (*p* < 0.01 versus control), but this effect was abrogated in dnRET-transfected neurons (*p* < 0.01 versus artefin alone; Figure 7A).

### ARTN- and Artefin-Induced Neurite Outgrowth Requires NCAM Expression

Although the above data clearly indicate that RET is involved in ARTN- and artefin-induced neurite outgrowth, other receptors may also be involved. The observed binding of ARTN and artefin to NCAM (Figure 4) suggests that NCAM may mediate some of the effects of ARTN and artefin. To investigate this possibility, we tested the neuritogenic potentials of ARTN and artefin in CGNs where NCAM expression was knocked down by transfection with NCAM shRNA, previously shown to be efficient (Hansen et al., 2007; Nielsen et al., 2009). In accordance, P2-d increased neurite outgrowth in mock-transfected neurons (*p* < 0.05), but this effect was eliminated in neurons transfected with NCAM shRNA (*p* < 0.01; Figure 7B). Mock-transfected CGNs stimulated with ARTN showed a significantly higher level of neurite outgrowth compared to unstimulated controls (*p* < 0.05; Figure 7B). However, down-regulation of NCAM expression completely abolished the neurite outgrowth effect of ARTN (Figure 7B), indicating that NCAM is involved in ARTN-induced neurite outgrowth. Similarly, artefin promoted the neurite outgrowth in mock-transfected neurons (*p* < 0.01), but this effect was abrogated in neurons where NCAM expression was knocked down (*p* < 0.001; Figure 7B). Thus, the neuritogenic effect of artefin, similar to the neuritogenic effect of ARTN, appears to be NCAM-dependent.

### ARTN- and Artefin-Induced Neurite Outgrowth Requires Activation of NCAM-Associated Signaling

To further investigate the role of NCAM in ARTN- and artefin-induced neurite outgrowth, we examined if signaling downstream of NCAM was activated. NCAM does not possess any intracellular catalytic activity and utilizes other molecules, particularly the FGFR, for induction of intracellular signaling (Francavilla et al., 2009). We stimulated CGNs with ARTN or artefin in the absence or presence of a pharmaceutical FGFR-inhibitor, SU5402, and observed the effect on neurite elongation.

**FIGURE 8:**
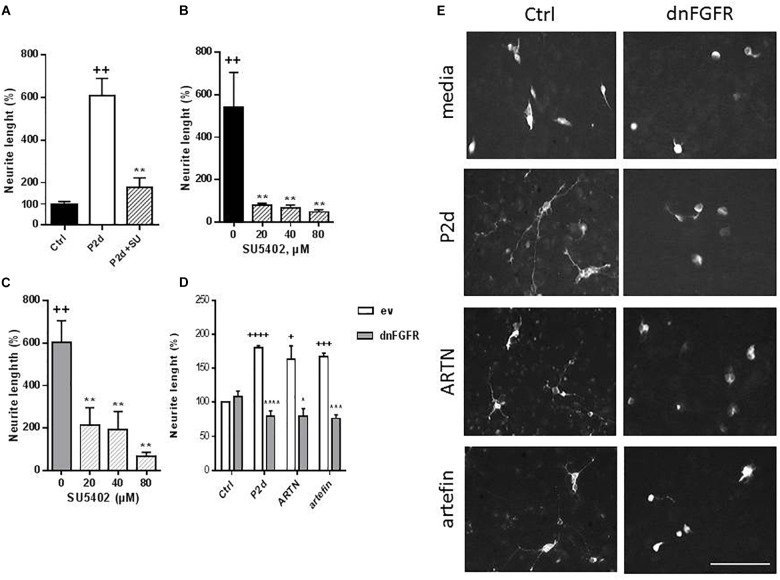
Inhibition of ARTN- and artefin-induced neurite outgrowth in CGNs by FGFR-inhibitor SU5402. **(A)** Neurite outgrowth induced by P2-d peptide (8 μg/ml). + Presents *p*-values for comparison of the neuritogenic effect of the peptide to the untreated neurons, while ^∗^ shows *p*-values for comparison to the positive control of CGNs stimulated with P2-d. **(B)** Inhibition of neurite outgrowth with serially diluted SU5402 (20, 40, 80 μM) induced by ARTN (0.21 nM) **(B)** or artefin (1.4 μM) **(C)**. ^∗^Shows *p*-values for comparison to the positive control of CGNs stimulated with ARTN or artefin. ++*p* < 0.01; ^∗∗^*p* < 0.01. **(D)** Inhibition of ARTN- and artefin-induced neuritogenic effect in dominant negative FGFR (dnFGFR) transfected CGNs. CGNs transfected with empty vector (ev) or dominant negative version of FGFR (dnFGFR) were plated and stimulated with P2-d (8 μg/ml), ARTN (0. 21 nM), or artefin (1.4 μM) for 24 h. The results are presented as percentage of control (CGNs transfected with empty vector) set at 100%, and the error bars indicate SEM. Student’s paired *t*-test was used for evaluation. +Presents *p*-values for comparison of the neuritogenic effect of compounds to the untreated neurons, while ^∗^ shows *p*-values for comparison to the positive control of CGNs transfected with empty vector. ^+^*p* < 0.05; ^+++^*p* < 0.001; ^++++^*p* < 0.0001; ^∗^*p* < 0.05; ^∗∗∗^*p* < 0.001; ^∗∗∗∗^*p* < 0.0001. **(E)** Micrographs of neurite outgrowth of CGNs transfected with dnFGFR and incubated with media alone, P2-d, ARTN, and artefin. Scale bar: 100 μm.

Treatment with P2-d, a known activator of FGFR downstream of NCAM (Soroka et al., 2002) promoted neurite outgrowth in the absence of SU5402 (*p* < 0.01), but co-incubation with 80 μM SU5405 significantly attenuated this effect (*p* < 0.01; Figure 8A).

The neurite outgrowth effect of ARTN was completely abolished in the presence of serially diluted inhibitor (*p* < 0.01; Figure 8B), indicating that ARTN-induced neurite outgrowth involves activation of the signaling downstream of NCAM.

Subsequent co-incubation of CGNs with SU5402 dose-dependently inhibited the neurite outgrowth effect of artefin (*p* < 0.01; Figure 8C). Therefore, artefin-induced neurite outgrowth seems to involve FGFR activation. To further confirm the role of NCAM-FGFR signaling, we transfected CGNs with dominant negative form of FGFR (Pankratova et al., 2016). The neurite outgrowth effect of ARTN and artefin was abolished in CGNs expressing dnFGFR (Figure 8D,E), confirming involvement of NCAM downstream signaling in the biological functions of ARTN and artefin.

## Discussion

The potential therapeutic use of neurotrophic factors have been intensively investigated ever since these factors were first identified (Rosenblad et al., 2000; Sariola and Saarma, 2003; Mannelli et al., 2011; Pallanti et al., 2014). For various reasons, however, neurotrophic factors have still not been successfully employed therapeutically. Mimetic compounds may overcome some of the obstacles connected with the use of neurotrophic factors, and in this study we applied a peptide-based approach to identify a new ARTN mimetic compound.

The study by Scott and Ibañéz (2001) demonstrated the cross-talk between GFLs and the members of GFRαs family. Their results showed that ARTN is the only GFL that can bind to all GFRαs, and the only one that can interact with GFRα3. According to solved structure of ARTN-GFRα3 complex, the extended epitopes located within Finger1 and 2 regions of ARTN are responsible for its interaction to GFRα3 (Wang et al., 2006), whereas the heel region in the ARTN molecule is involved in formation and maintenance of the dimer (Baloh et al., 2000b; Wang et al., 2004). Surprisingly, our results show that artefin (which corresponds to the heel region of ARTN) binds to GFRα3 although with lower affinity compared to ARTN. Moreover, artefin has biological functions in terms of CGN survival and neuritogenesis, where GFRα3-RET system is centrally involved. The biological function of artefin is determined by the specific amino acid sequence (because peptides with scrambled and reverse sequences are not biologically active), and it forms a functional structure that can bind and activate the receptor complex. Further profound computational analysis, like molecular dynamic simulation, would be required to identify the binding site of artefin motif on GFRα3. The study of neurite outgrowth demonstrates that ARTN and artefin possess strong neuritogenic potential that only requires interaction between growth factor or peptide and the receptor on the surface of the cell membrane. When the parent protein and the peptide are placed in the same cellular system, ARTN competes with artefin for binding to receptor complex. This might explain the inhibitory effect of high none-neuritogenic concentrations of ARTN on the neurite outgrowth induced by artefin. Our results suggest that ARTN and artefin use the same signal system for promoting neurite outgrowth. Based on our results, we speculate that in addition to GFRα3-RET, NCAM-FGFR could be an alternative signaling system mediating the biological effects of ARTN/artefin.

It has been determined that GFRα receptors and RET do not show overlapping expression in different brain regions (Nosrat et al., 1997; Trupp et al., 1997; Yu et al., 1998). It has been suggested that GDNF binds NCAM and that this interaction is potentiated by GFRα1 (Paratcha et al., 2003). RET-independent GDNF signaling also acts in neurons expressing RET, as evidenced by Chao et al. (2003), who found that NCAM antibodies suppressed GDNF-induced survival and differentiation of midbrain dopaminergic neurons. Whether NCAM can be an alternative receptor for other GFLs has been established by Paratcha et al. (2003), who showed that other GFLs can bind to NCAM-containing complexes with their corresponding GFRα. This suggests that ARTN and other GFLs may also utilize NCAM as a receptor. Notably, while GFLs do not bind to RET in the absence of their cognate GFRα (Anders et al., 2001), GDNF interacts directly with NCAM (Paratcha et al., 2003; Nielsen et al., 2009). NCAM could thus function as a signaling receptor for ARTN through a direct interaction between ARTN and NCAM. Although, we do not exclude the possibility, that knocking down of NCAM might affect the expression of other, yet unidentified, proteins potentially involved in mediation of ARTN effects, and thus the effect of NCAM knock-down on ARTN-induced neurite outgrowth might be indirect. To the best of our knowledge, there are no data confirming RET-independent ARTN-induced signaling. Our experiments show that inhibitory RET antibody can interfere with ARTN- and artefin-induced neurite outgrowth but does not completely block it, suggesting that some other receptor plays a role in ligand-induced neurite elongation. The absence of significant neurite outgrowth in NCAM knock down and dnFGFR CGNs further supports this suggestion (Figure 7B, 8D). Furthermore, we did not observe ARTN- and artefin-induced neurite outgrowth in neurons expressing dominant negative RET (Figure 7A), suggesting that both NCAM and RET are necessary for ARTN-induced signaling in CGNs. We speculate that the presence of NCAM in the signal system is always obligatory, but that it may be an alternative receptor for ARTN together with RET in the CGNs model.

Interestingly, artefin showed much stronger neurite outgrowth effect than ARTN. It is not unusual for a peptide mimetic, representing a small region of the molecule, to show a stronger efficacy than the whole molecule but to be less potent (as it needs a higher concentration to be effective) (Gjoerlund et al., 2012). The parent growth factor is typically more specific than small peptides and may contain spatially distant domains that can modulate the effect of the whole molecule. As artefin is synthesized as dendrimer consisting of four monomers coupled to a lysine backbone, one peptide can potentially bind four receptors simultaneously, this gives a stronger effect. Moreover, as we showed, this peptide keeps its α-helical conformation in solution, thus perfectly mimicking the binding interface in the ARTN molecule (Figure 1B). We suggest that the secondary structure of artefin is important for the functioning of this peptide mimetic. Further studies are required to elucidate the possible mechanisms.

## Conclusion

We confirmed that ARTN binds and signals through GFRa3-RET complex. We also found that ARTN bound directly to NCAM and that NCAM expression and activation of its downstream signaling partner, FGFR, were required for ARTN-induced neuritogenesis, thereby indicating that NCAM is an alternative receptor for ARTN. We propose that the heel region within the ARTN is a potential binding site of ARTN to NCAM. The peptide, derived from this heel region, artefin, has neuroprotective and neurite outgrowth effects similar to those of the parent growth factor, it is more effective but less potent. Both ARTN and artefin act through the same signal system(s), but the growth factor has a higher binding affinity. Further comprehensive study including mutagenesis is needed to identify the biological function(s) of heel region of ARTN.

## Note

To the memory of Vladimir Berezin, deceased February 14, 2016.

## Bioethics

Animals were treated in accordance with the Danish Animal Welfare Act, and the study was approved by the Department of Experimental Medicine at the University of Copenhagen.

## Ethics Statement

All experiments were performed according to European Union legislation and with licenses from the Danish Animal Experiments Inspectorate. All animals were kept under standard conditions (23°C, 50% humidity, 12-h light/dark cycle) with free access to food and water. The number of animals utilized in the respective experiments was kept to a minimum, and all work was conducted in a manner designed to cause the least harm and suffering to the animals.

## Author Contributions

MI, JN, IK, KG, SP, and EB conceived and designed the experiments. MI, JN, IK, KG, and SP performed the experiments. MI, JN, SP, EB, and TM analyzed the data and wrote the manuscript. EB and TM directed and supervised the project.

## Conflict of Interest Statement

The authors declare that the research was conducted in the absence of any commercial or financial relationships that could be construed as a potential conflict of interest.

## Supplementary Material

FIGURE S1 The scheme of dendrimeric artefin peptide. The figure shows a line-angle representation of the structure of the lysine backbone with four artefin sequences (RSPHDLSLALLGAG) attached.

FIGURE S2 Surface plasmon resonance analysis of binding affinity of **(A)** artefin to GFRα1 and **(B)** artefin to GFRα2.
