# Supplementary figures and images for: Artemin and an Artemin-Derived Peptide, Artefin, Induce Neuronal Survival, and Differentiation Through Ret and NCAM
Source: Front Mol Neurosci. 2019 Feb 22;12:47. doi: 10.3389/fnmol.2019.00047 (PMC6396024; doi:10.3389/fnmol.2019.00047)

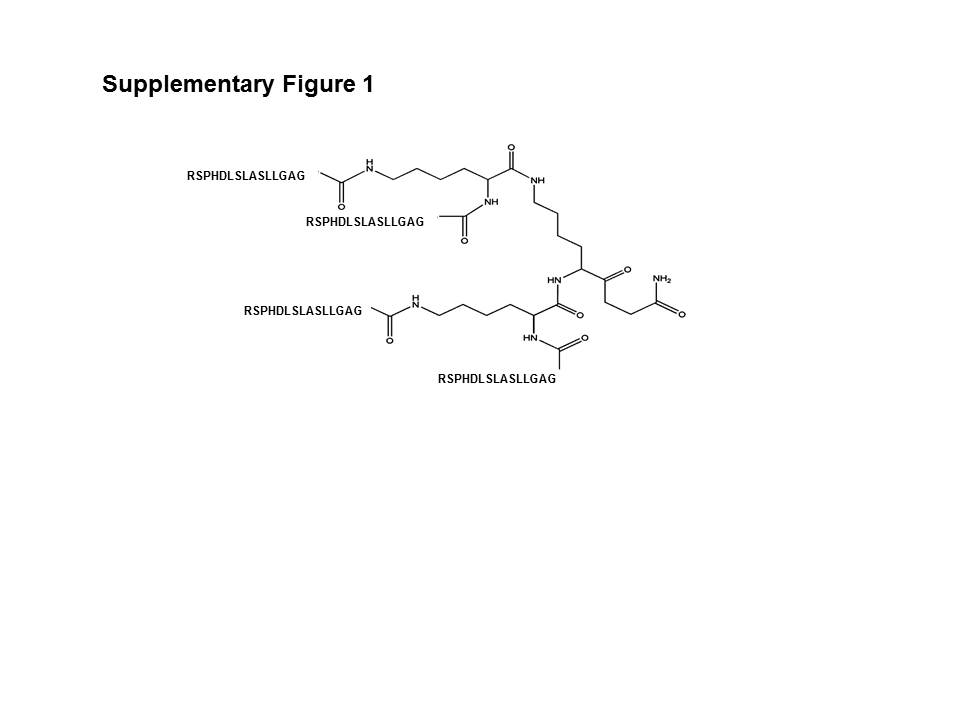

Supplement: FIGURE S1 — The scheme of dendrimeric artefin peptide. The figure shows a line-angle representation of the structure of the lysine backbone with four artefin sequences (RSPHDLSLALLGAG) attached. [file Image_1.jpg]

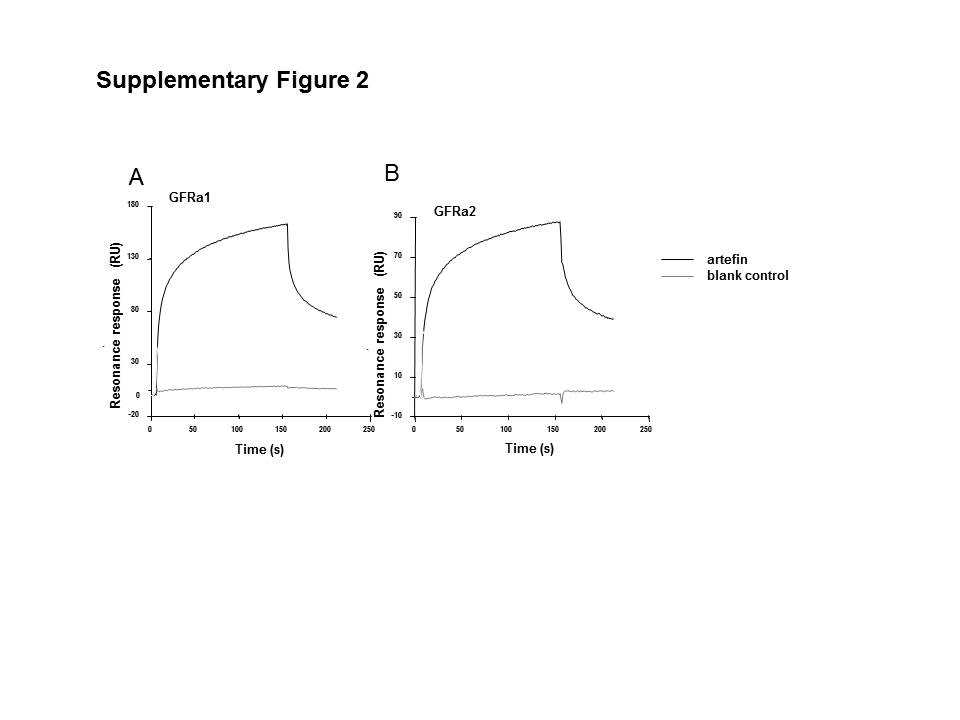

Supplement: FIGURE S2 — Surface plasmon resonance analysis of binding affinity of (A) artefin to GFRα1 and (B) artefin to GFRα2. [file Image_2.JPEG]
